# Hypoxia‐activated fluorescent probes as markers of oxygen levels in plant cells and tissues

**DOI:** 10.1111/nph.70202

**Published:** 2025-05-08

**Authors:** Monica Perri, M. Shahneawz Khan, Antoine L. D. Wallabregue, Viktoriia Voloboeva, Amber M. Ridgway, Edward N. Smith, Hannah Bolland, Ester M. Hammond, Stuart J. Conway, Daan A. Weits, Emily Flashman

**Affiliations:** ^1^ Department of Biology University of Oxford South Parks Road Oxford OX1 3RB UK; ^2^ Department of Chemistry, Chemistry Research Laboratory University of Oxford Mansfield Road Oxford OX1 3TA UK; ^3^ Experimental and Computational Plant Development, Institute of Environment Biology Utrecht University Padualaan 8 Utrecht 3584 CH the Netherlands; ^4^ PlantLab Institute of Plant Sciences Scuola Superiore Sant'Anna 56010 Pisa Italy; ^5^ Department of Oncology University of Oxford Old Road Campus Research Building Oxford OX3 7DQ UK; ^6^ School of Veterinary Medicine, Faculty of Health and Medical Sciences University of Surrey Guildford Surrey GU2 7AL UK; ^7^ Department of Chemistry & Biochemistry University of California Los Angeles 607 Charles E. Young Drive East, PO Box 951569 Los Angeles CA 90095‐1569 USA

**Keywords:** bioreduction, fluorogenic probes, hypoxia, oxygen‐sensing, Resorufin

## Abstract

- Low oxygen signalling in plants is important in development and stress responses. Measurement of oxygen levels in plant cells and tissues is hampered by a lack of chemical tools with which to reliably detect and quantify endogenous oxygen availability. We have exploited hypoxia‐activated fluorescent probes to visualise low oxygen (hypoxia) in plant cells and tissues.
- We applied 4‐nitrobenzyl (4NB‐) resorufin and methyl‐indolequinone (MeIQ‐) resorufin to *Arabidopsis thaliana* whole cells and seedlings exposed to hypoxia (1% O_2_) and normoxia (21% O_2_). Confocal microscopy and fluorescence intensity measurements were used to visualise regions of resorufin fluorescence.
- Both probes enter *A. thaliana* whole cells and are activated to fluoresce selectively in hypoxic conditions. Similarly, incubation with *A. thaliana* seedlings resulted in hypoxia‐dependent activation of both probes and observation of fluorescence in hypoxic roots and leaf tissue. MeIQ‐Resorufin was used to visualise endogenous hypoxia in lateral root primordia of normoxic *A. thaliana* seedlings.
- Oxygen measurement in plants until now has relied on invasive probes or genetic manipulation. The use of these chemical probes to detect and stain applied and endogenous hypoxia has the potential to facilitate a greater understanding of oxygen concentrations in plant cells and tissues, allowing the correlation of oxygen availability with acclimative and developmental responses to hypoxia.

## Abstract 2

See also the Commentary on this article by Pedersen, **247**: 2492–2494.

## Introduction

Plants require oxygen for the production of ATP and other important biomolecules and rely on photosynthesis and diffusion or convection to deliver oxygen to cells and tissues. Regions of low oxygen and oxygen gradients are common in plants (Weits *et al*., 2021) due to high metabolic oxygen consumption, as well as physical barriers to diffusion. The latter arise from hydrophobic and waxy layers present at the surfaces of some tissues, which prevent gas diffusion, including in legume root nodules where anaerobic conditions are critical for effective nitrogen fixation (Venado *et al*., 2022), the cuticle and suberised cell layers in seeds in which low oxygen is an important developmental cue (Borisjuk & Rolletschek, 2009; Langer *et al*., 2023) and the deposition of suberin at the surfaces of adventitious roots formed to prevent radial oxygen loss (Peralta Ogorek *et al*., 2024). For example, oxygen concentrations of < 1% are reported in seeds (Borisjuk & Rolletschek, 2009). Shoot apical meristems and lateral root primordia have metabolically driven low O_2_ tensions of *c*. 3% and < 5% O_2_, respectively (Shukla *et al*., 2019; Weits *et al*., 2019), and O_2_ gradients across *Ricinus communis* (castor bean) stems can range from 21% at the surface to 7% in the vascular tissue (van Dongen *et al*., 2003). In addition to endogenous variations in O_2_ tension, plants can be exposed to hypoxia (where oxygen is at a lower concentration than physiologically typical) through submergence (Bailey‐Serres *et al*., 2012) and pathogen infection; *Botrytis cinerea*‐infected Arabidopsis leaves and crown gall tumours arising from the infection of wounded tissue by *Agrobacterium tumefaciens* result in O_2_ tensions of 2–5% (Kerpen *et al*., 2019; Valeri *et al*., 2021).

Molecular pathways that drive adaptive responses to hypoxia or molecular outcomes of low oxygen niches are well understood (Weits *et al*., 2021); however, the intracellular oxygen concentrations that trigger these responses are not accurately known. To date, it has been challenging to accurately quantify oxygen levels in plant cells and tissues noninvasively (Akter *et al*., 2021): The most widely used method of oxygen measurement is the Clark‐type electrode, which measures oxygen concentrations at distinct locations (Geigenberger *et al*., 2000; Colmer *et al*., 2020). Mini electrodes enable resolution to 10 μm and can reveal cross‐sections of O_2_ gradients across tissues (Weits *et al*., 2019), but the electrodes are invasive (thus creating physical damage) and also consume O_2_ locally. Luminescence quenching‐based probes have also been used but are similarly invasive and can be impacted by other quenching agents (Papkovsky, 1993; Pedersen *et al*., 2016; Mori *et al*., 2019; Akter *et al*., 2021). A number of biosensor cassettes have been applied to measure oxygen levels *in vivo* without this invasive treatment (reviewed in Weits *et al*., 2021). These have exploited a heterologous mammalian O_2_‐sensing system to drive luciferase activity (Iacopino *et al*., 2019) and used a hypoxia‐responsive promoter element (HRPE) to drive the expression of dual fluorescence proteins (Panicucci *et al*., 2020). Although effective, these strategies require significant engineering and are not easily applicable to a range of plant tissues or species. There are no chemical probes reported to date applicable in plants for O_2_ sensing. Since they do not require genetic manipulation or invasive procedures, chemical probes represent a promising system to investigate oxygen sensing in plant cells and shed light on physiological responses to these conditions. An ideal oxygen‐sensing probe should have high sensitivity to hypoxia, potentially finely discriminating among oxygen levels, should be nontoxic, permeable to plant cells, detectable in plant systems and potentially reversible to accurately reflect real‐time oxygen dynamics. Considering that the structure of an elaborate cell wall strictly limits the uptake of large molecules in plants (Tepfer & Taylor, 1981), efficient tissue permeability needs to be tested for chemical probes. In the case of fluorescence‐based chemical probes, avoiding fluorescence spectral overlap between probe and Chl is also a prerequisite consideration for such O_2_ sensing in plants.

**Fig. 1:**
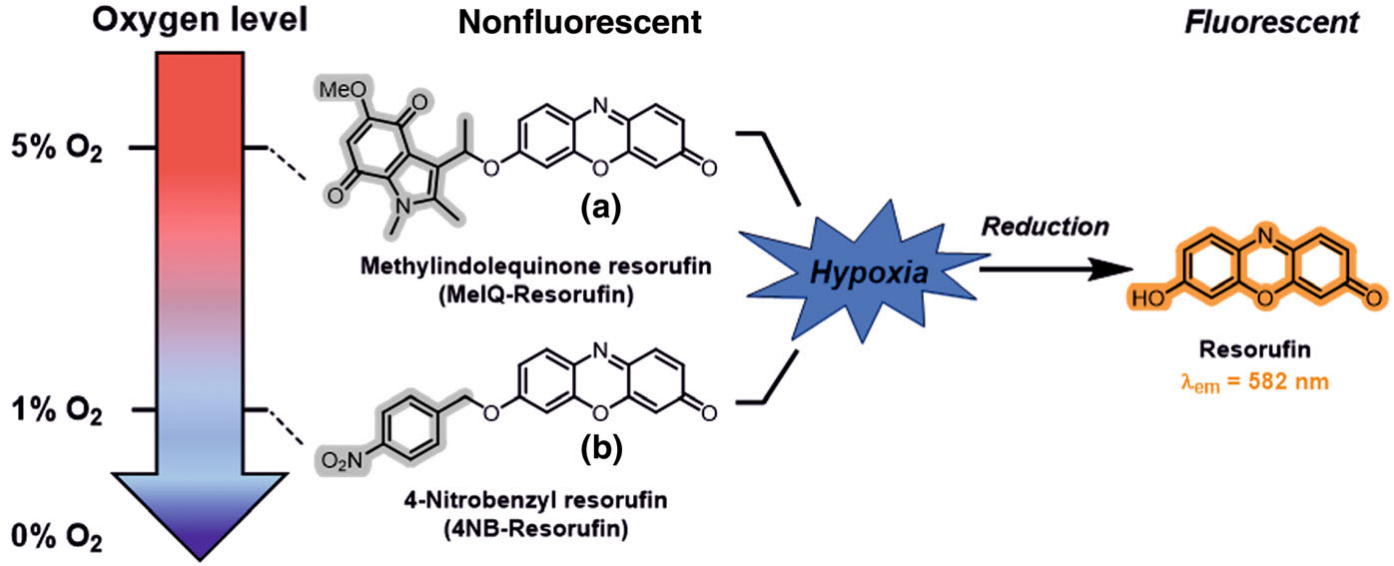
Schematic representation of bioreductive hypoxia‐activated chemical probes. Methyl‐indolequinone resorufin (MeIQ‐Resorufin, a) and 4‐nitrobenzyl resorufin (4NB‐Resorufin, b): reduction under hypoxic conditions liberates fluorogenic resorufin.

Hypoxia‐activated imaging agents have been used extensively in mammalian cell and tissue biology to visualise intracellular hypoxia via fluorescence or immunostaining (Nordsmark *et al*., 2003; Koch, 2008; Elmes, 2016; Close & Johnston, 2022). These chemical tools exploit the oxygen‐sensitive activity of reductase enzymes: In low oxygen, these enzymes can catalyse bioreduction in redox‐sensitive functional groups to generate products that can be visualised. One such example is 4‐nitrobenzene‐resorufin (4NB‐Resorufin); hypoxia‐dependent bioreduction of 4NB‐Resorufin results in the cleavage of the 4NB group to release fluorescent resorufin (Fig. 1). The proposed mechanism for the bioactivation of resorufin‐based probes in mammalian and bacterial systems relies on the fact that the attachment of the 4‐nitrobenzyl group to the phenol of resorufin results in a nonfluorescent compound. In normoxia, nitroreductase catalyses one‐electron reduction of the nitro group, but the compound is rapidly back‐oxidised and the resorufin remains uncleaved; however, further reduction in the group occurs under hypoxic conditions, leading to the release of the resorufin fluorophore (Collins *et al*., 2018). Bioreductive moieties such as the nitroaryl groups, quinones and *N*‐oxides have been used in hypoxia‐activated prodrugs in mammalian systems (McKeown *et al*., 1995; O'Connor *et al.,* 2016; Sharma *et al*., 2019; Skwarska *et al*., 2021; Tosun *et al*., 2023). Additionally, it was shown that isopropyl β‐d‐1‐thiogalactopyranoside (IPTG) in conjugation with the 4‐nitrobenzene moiety is inactive, but hypoxia‐dependent activation of the molecule releases the IPTG that induces the expression of the GFP protein in bacteria (Collins *et al*., 2018).

We hypothesised that hypoxia‐activated fluorescent probes could be applied to plant cells and tissues as a way to visualise low oxygen conditions and thus contributing to a clearer understanding of oxygen signalling processes in plants. We therefore investigated two resorufin‐based fluorogenic probes, 4NB‐Resorufin and a recently reported methyl‐indolequinone‐conjugated resorufin probe (MeIQ‐Resorufin, which has been used in human cancer cell lines; Wallabregue *et al*., 2023) to determine whether they could permeate plant cells and tissues to report on low oxygen conditions. 4NB‐Resorufin has been reported to permeate bacterial cells (Collins *et al*., 2018); therefore, we tested whether this probe might also be able to cross plant cell walls. MeIQ‐Resorufin has recently been reported to be activated at higher oxygen concentrations than 4NB‐Resorufin (Wallabregue *et al*., 2023); therefore, we also tested the possibility that this probe could be applied to plants, and in combination, the probes could differentiate oxygen concentrations in *Arabidopsis thaliana* cell and tissue cultures. Our results reveal that these hypoxia‐activated probes are effective at visualising low oxygen conditions (herein termed hypoxia) in this model plant, thus paving the way for an expanded toolkit in detecting and measuring hypoxic conditions in plants.

## Materials and Methods

### MeIQ‐Resorufin and 4NB‐Resorufin synthesis

MeIQ‐Resorufin and 4NB‐Resorufin were synthesised as described previously (Wallabregue *et al*., 2023; Collins *et al*., 2018). To yield MeIQ‐Resorufin, diisopropyl azodicarboxylate was added dropwise to a solution of 3‐(1‐hydroxyethyl)‐5‐methoxy‐1,2‐dimethyl‐1*H*‐indole‐4,7‐dione, resorufin and triphenylphosphine in anhydrous tetrahydrofuran at room temperature under argon. The reaction mixture was stirred vigorously for 24 h at 50°C, then concentrated under reduced pressure. The resulting residue was dissolved in ethyl acetate, washed with aqueous sodium hydrogen carbonate, water, aqueous hydrochloric acid (1 M solution) and brine, dried (sodium sulphate), filtered and concentrated under reduced pressure. The crude material was purified using silica gel flash column chromatography. The residue was dissolved in ethyl acetate and precipitated by adding hexane. The precipitation procedure was repeated twice. The precipitate was then dried under reduced pressure to give the title compound as an orange solid. The timescale for this synthesis is *c*. 3 wk.

To prepare 4NB‐Resorufin, 4‐nitrobenzylbromide in *N*,*N*‐dimethylformamide was added to a solution of resorufin and potassium carbonate, stirred at 40°C for 3 h and then cooled to room temperature. The solution was diluted with ethyl acetate and washed with brine. The aqueous layer was extracted with ethyl acetate. The organic components were combined, dried (sodium sulphate), filtered and concentrated under reduced pressure. The crude residue was recrystallised from dichloromethane, followed by trituration with diethyl ether to yield the title compound as a red solid. Further details are provided in the Methods S1. All the probes were dissolved in dimethyl sulfoxide (DMSO) to reach a stock concentration of 1 mM and stored at −20°C in black Eppendorf tubes to avoid light penetration. The timescale for this synthesis is *c*. 1–2 d.

### Plant materials and growth conditions

*Arabidopsis thaliana* (L.) Heynh. Columbia (Col‐0) ecotype was used for seedlings. For *in vitro* propagation, sterilised seeds were cultivated on ½‐strength Murashige & Skoog medium (½MS; Murashige & Skoog, 1962) supplemented with 1% (w/v) sucrose and 0.8% (w/v) agar, and grown vertically at 22°C, 16 h : 8 h, day : night photoperiod 100 μmol photons m^−2^ s^−1^. Two‐week‐old seedlings were used for the experiments. *Arabidopsis thaliana Landsberg erecta* ecotype was used for whole cell cultures; these were propagated in full‐strength MS medium, supplemented with 3% (w/v) glucose, 2.5 μg ml^−1^ 1‐naphthaleneacetic acid (NAA) and 0.25 μg ml^−1^ kinetin adjusted to pH 5.8, and renewed weekly. Experiments were performed with cells from cultures, which had been growing for 4 d following subculture.

### Probe incubation

For incubation with cell cultures, 1 ml of 4‐d‐old cell culture was incubated with a final concentration of 10 μM of probe for 2 h (based on studies with similar probes in mammalian cells (Wallabregue *et al*., 2023) as well as time‐course studies of fluorescence intensity in the presence of each probe; Fig. 3). One millilitre of cell cultures incubated with 1% DMSO and 1 ml of MS incubated with either 1% DMSO or 10 μM of probe were used as controls. For seedlings treatment, 2‐wk‐old *A. thaliana* seedlings were incubated with 1 ml of liquid MS medium supplemented with 100 μM of probe unless otherwise mentioned for 6 h to allow time for tissue entry and diffusion. Seedlings incubated with 10% DMSO, MS incubated with the same amount of probe or DMSO, were used as negative controls. All the experiments were performed using 12‐well plates, with three biological replicates per plate unless otherwise stated. Each biological replicate is represented by 1 ml of Arabidopsis cell suspension or one 2‐wk‐old seedling for the cell culture and seedling experiments, respectively.

### Hypoxia treatment

All the hypoxic treatments were conducted using a CLARIOstar® plate reader (BMG LabTech, Ortenberg, Germany) supplemented with an atmospheric control unit (ACU) to regulate O_2_ gas level. Probe sensitivity to hypoxia was evaluated at 5, 1, 0.5, 0.3 and 0.2% O_2_, using the ACU, flushed with N_2_ (g), for 2 h (cell culture treatment) or 6 h (seedlings), in the dark; the ACU was turned off for controls under normoxic conditions. Plates were shaken at 100 rpm every 30 s before fluorescence detection. For each cycle, 20 readings per well were taken and the mean was calculated. Resorufin was detected upon excitation at 550 nm and emission at 585 nm, using a dichroic filter of 567.5 nm. Two‐week‐old *A. thaliana* Col‐0 seedlings expressing a HRPE:nLUC construct (Akter *et al*., 2024) were used for *in vivo* luciferase detection upon treatment under normoxic or hypoxic conditions (1% O_2_ v/v O_2_/N_2_), for 1 h in the dark, using the CLARIOstar® plate reader (BMG LabTech). Four biological replicates were employed in the experiment, and wild‐type genotype was used as a control. Luminescence was detected using the Nano‐Glo® Vivazine™ live cell substrate (Promega), following the manufacturer's instruction. Measurements were collected for 20 min, seven measurements per minute were taken, and the mean was calculated.

### Confocal imaging

Four‐day‐old *A. thaliana* cell cultures and 2‐wk‐old seedlings were used for probe detection after hypoxic treatment (1% O_2_ v/v O_2_/N_2_) for 2 h (cell culture) or 6 h (seedlings). For propidium iodide (PI) staining, cell cultures were incubated with 10 μg ml^−1^ of PI solution for 5 min, then mounted on slides. Imaging was performed using either ZEISS LSM 780 (Micron, Department of Biochemistry, University of Oxford) or ZEISS LSM880 Airyscan microscope (Department of Biology, University of Oxford), equipped with a 25D7 objective lens, upon laser excitation at 561 nm and collection at 570–590 nm for Resorufin imaging, excitation at 488 nm and collection at 604–622 nm for PI, and excitation at 561 nm and collection at 650–750 nm for Chl. For lateral root primordia, *A. thaliana* seedlings were grown on ½MS supplemented with 0.5% sucrose for 7 d and then incubated with 100 μM MeIQ‐Resorufin for 2 h in 6‐well plates under normoxic (21% O_2_) or hypoxic (1% O_2_) conditions. After washing in distilled water for 10 min, seedlings were imaged using a Zeiss LSM800 Airyscan laser scanning confocal microscope (University of Utrecht) with 561‐nm excitation laser light and 565–616 nm emission detection. Fluorescence intensity was calculated using the ImageJ software (Schindelin *et al*., 2012). Six measurements were taken for each image, divided between background and signal (three measurements per category). The signal intensity was quantified as the ratio of the mean signal measurement to the mean of the background measurements for each image. This process was repeated for three images per probe, under each experimental condition.

### Root length and fresh weight measurements

To measure plant tolerance to the probe, *A. thaliana* seedlings were grown vertically on squared plates for 7 d and then treated with 100 μM 4NB‐Resorufin, MeIQ‐Resorufin or resorufin, or 10% DMSO, as a negative control, under air and 16 h : 8 h photoperiod conditions. After 4 d of treatment, plates were scanned using an EPSON Perfection V750 PRO scanner with a resolution of 720 dots per inch. Primary root length was assessed using ImageJ (Schindelin *et al*., 2012). Twenty‐one to twenty‐eight biological replicates were used for root length measurements. Growth rate per day was assessed as the increase in the length of the primary root after 4 d of treatment compared with the beginning of treatment. Fresh weight was measured after 4 d of treatment using four biological replicates, each consisting of seven whole seedlings.

### Spectroscopy

Fluorescence spectra were recorded using a HORIBA Jobin Yvon FluoroLog3 fluorimeter (Hamamatsu R928 detector and a double‐grating emission monochromator). The standard conditions for acquiring emission and excitation spectra are room temperature and steady‐state mode unless otherwise stated. Fluorescence spectra were obtained by using the GraphPad Prism software.

Ultraviolet‐visible (UV‐Vis) spectra were recorded on a V‐770 UV–Visible/NIR Spectrophotometer equipped with a Peltier temperature controller and stirrer using disposable polystyrene cuvettes of 1 cm path length. Experiments were conducted at 25°C unless otherwise stated. Ultraviolet‐visible spectra were plotted using the GraphPad software. pH measurements of solutions were taken using an Oakton pH meter.

### Reactive oxygen species preparation and assays

#### General procedure for hydrogen peroxide (H_2_O_2_ ) assay

To a quartz cuvette on ice (0–4°C) containing Milli‐Q water and H_2_O_2_ solution, a probe solution was added. The cuvette was sealed and left at room temperature for 5 min. At this point, the final concentrations of H_2_O_2_ and the hypoxia‐imaging probe were 100 and 1 μM, respectively. The fluorescence spectra were then measured using the specified parameters at the given time point. Further details are provided in the Methods S1.

#### General procedure for hydroxyl radical (HO·) assay

##### Preparation

The hydroxyl radical (HO·) was generated using the Fenton reaction. Ferrous chloride (1 eq.) was added to a solution of H_2_O_2_ (10 eq.). Upon mixing, the solution turned orange and was used immediately for analysis.

##### Assay

To a quartz cuvette on ice (0–4°C) containing Milli‐Q water and generated HO· solution, probe solution was added. At this stage, the final concentrations of HO· and the hypoxia‐imaging probe were 100 and 1 μM, respectively. The solution was homogenised by pipetting for 10 s, and then, the cuvette was sealed and left at room temperature for 5 min. Fluorescence spectra were measured under the specified parameters. Further details are provided in the Methods S1.

### General procedure for light stability assay

To a vial containing DMSO, a probe solution was added. An aliquot was taken from the reaction solution and transferred to a quartz cuvette, followed by the addition of Milli‐Q water. The solution was homogenised by pipetting for 10 s; then, the fluorescence spectra were recorded using the specified parameters, serving as the initial time point (T = 0, where T represents time).

The vial was then sealed and placed in an aluminium box, positioned 20 cm away from a white LED and illuminated for 4 h. After illumination, another aliquot was collected, transferred to a quartz cuvette and diluted with Milli‐Q water. The fluorescence spectrum was subsequently recorded under the specified parameters. Further details are provided in the Methods S1.

### General procedure for the assay with different pH or ionic strength buffers

To a quartz cuvette containing buffer with the desired pH or NaCl concentration, a probe solution was added. The solution was homogenised by pipetting for 10 s, and then, the cuvette was sealed and left at room temperature for 10 min (NaCl), and 5 or 60 min (pH buffers). The fluorescence spectra were then recorded using the specified parameters. Further details are provided in the Methods S1.

### Statistical analyses

Statistical analyses were performed using the GraphPad Prism 10.3.0 or R Statistical Software (v.4.1.3; Foundation for Statistical Computing, Vienna, Austria). Normal distribution of data was evaluated through the Shapiro–Wilk test. Based on the outcome of the normality test, the appropriate tests were performed to evaluate differences. Additional details are provided in the legend of the corresponding figure.

## Results

### 4NB‐Resorufin and MeIQ‐Resorufin can permeate *Arabidopsis thaliana* whole cells and show hypoxia‐dependent activation

We first set out to determine whether the 4NB‐ and MeIQ‐Resorufin probes could permeate *A. thaliana* cells. Four‐day‐old whole autotrophic suspension‐cultured cells were incubated with either 10 μM probe or 1% DMSO loading control before shaking in the dark for 2 h under normoxia (21% O_2_) or hypoxia (1% O_2_). Cells were visualised using a confocal microscopy to detect fluorescence arising from resorufin (λ_ex/em_ 561/570–590 nm), Chl (λ_ex/em_ 561/650–750 nm) and PI (λ_ex/em_ 488/604–622 nm). Propidium iodide staining was exploited for live/dead cell imaging, since this dye is not cell‐permeable in intact membranes (Riccardi & Nicoletti, 2006).

**Fig. 2:**
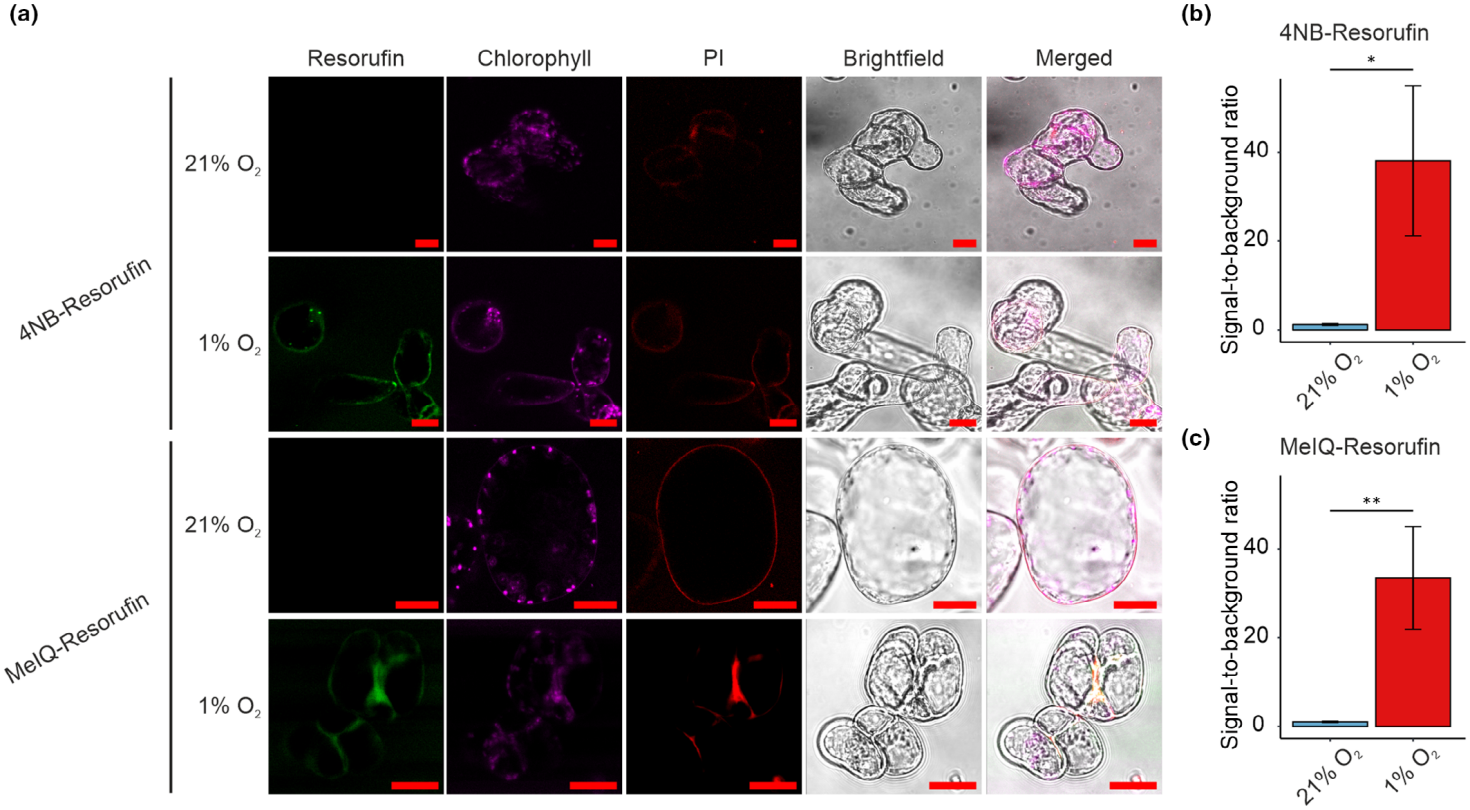
Hypoxic incubation of *Arabidopsis thaliana* cells with 4NB‐Resorufin and MeIQ‐Resorufin results in fluorescence. (a) One millilitre *A. thaliana* whole cells were incubated with 10 μM 4‐NB‐Resorufin or MeIQ‐Resorufin at 21% or 1% O_2_ for 2 h. Cells were examined for fluorescence of resorufin (570–590 nm), Chl (650–750 nm) and propidium iodide (PI, 604–622 nm). Images show representative cells from ≥ 3× wells containing 1 ml of cells. Bars, 20 μm. (b, c) Quantification of resorufin fluorescence intensity in cells treated with 4NB‐ or MeIQ‐Resorufin upon hypoxia or normoxia represented by the ratio of the signal intensity to the background (*n* ≥ 3). Error bars show standard deviation; statistical significance was determined using two‐sided Student's *t*‐test (*, *P*‐value < 0.05; **, *P*‐value < 0.01).

The arising images (Fig. 2a, Supporting Information Fig. S1) and signal quantification (Fig. 2b) show that when cells were incubated with either 4NB‐Resorufin or MeIQ‐Resorufin in hypoxic conditions, a significant increase in fluorescence in the 570–590 nm range was observed in cytosolic regions of the cell, consistent with resorufin fluorescence (Fig. 2b,c). No fluorescence was observed at this wavelength range when cells were incubated with either 4NB‐Resorufin or MeIQ‐Resorufin in normoxia (Fig. 2a). Similarly, no fluorescence was observed if cells were incubated in hypoxia and treated with 1% DMSO only, nor if either probe was incubated in MS media, in hypoxia and in the absence of cells (Fig. S2). Chl autofluorescence was also observed in the presence of both 4NB‐Resorufin and MeIQ‐Resorufin and when cells had been incubated in both normoxia and hypoxia. PI fluorescence was used to demonstrate that the probes were entering cells and that the cells remained viable. Propidium iodide fluorescence emission was only observed at cell surfaces, indicating the probes were not toxic to the cell under the conditions tested. Propidium iodide staining was then used to determine the proportion of dead or damaged cells as a function of increasing concentrations of each probe (Fig. S3a,b). Across the different concentrations tested, there was no significant increase in PI staining with increasing concentration of probe, relative to DMSO control. There was some increase in PI staining in hypoxic control cells compared with normoxic control cells, although this was not significant. Overall, the results indicate that, under the conditions used, the presence of the probes does not cause significant damage to the cell membranes and is no more damaging to cells than hypoxia itself.

The results therefore indicate that both probes are able to permeate Arabidopsis whole cells and enter the cytoplasm. The observed fluorescence in hypoxia but not in normoxia indicates that the probes have been bioreductively cleaved to form resorufin in a manner that is dependent on a sustained decrease in oxygen availability. This is consistent with the bioreductive properties of these probes in bacterial and mammalian systems (Collins *et al*., 2018; Wallabregue *et al*., 2023). Although the reductase enzyme(s) responsible for bioreduction in the probes have not been identified in plants, the same probes are reduced by nitroreductase and CYP450 enzymes in mammalian and bacterial cells (O'Connor *et al*., 2017; Collins *et al*., 2018; Wallabregue *et al*., 2023), suggesting that similar enzymes may be enabling probe activation in plant cells. These probes are therefore able to detect whether whole plant cells grown in culture are hypoxic.

### Quantitative fluorescence detection of hypoxia‐dependent bioreduction in 4NB‐Resorufin and MeIQ‐Resorufin in a plate‐based format

**Fig. 3:**
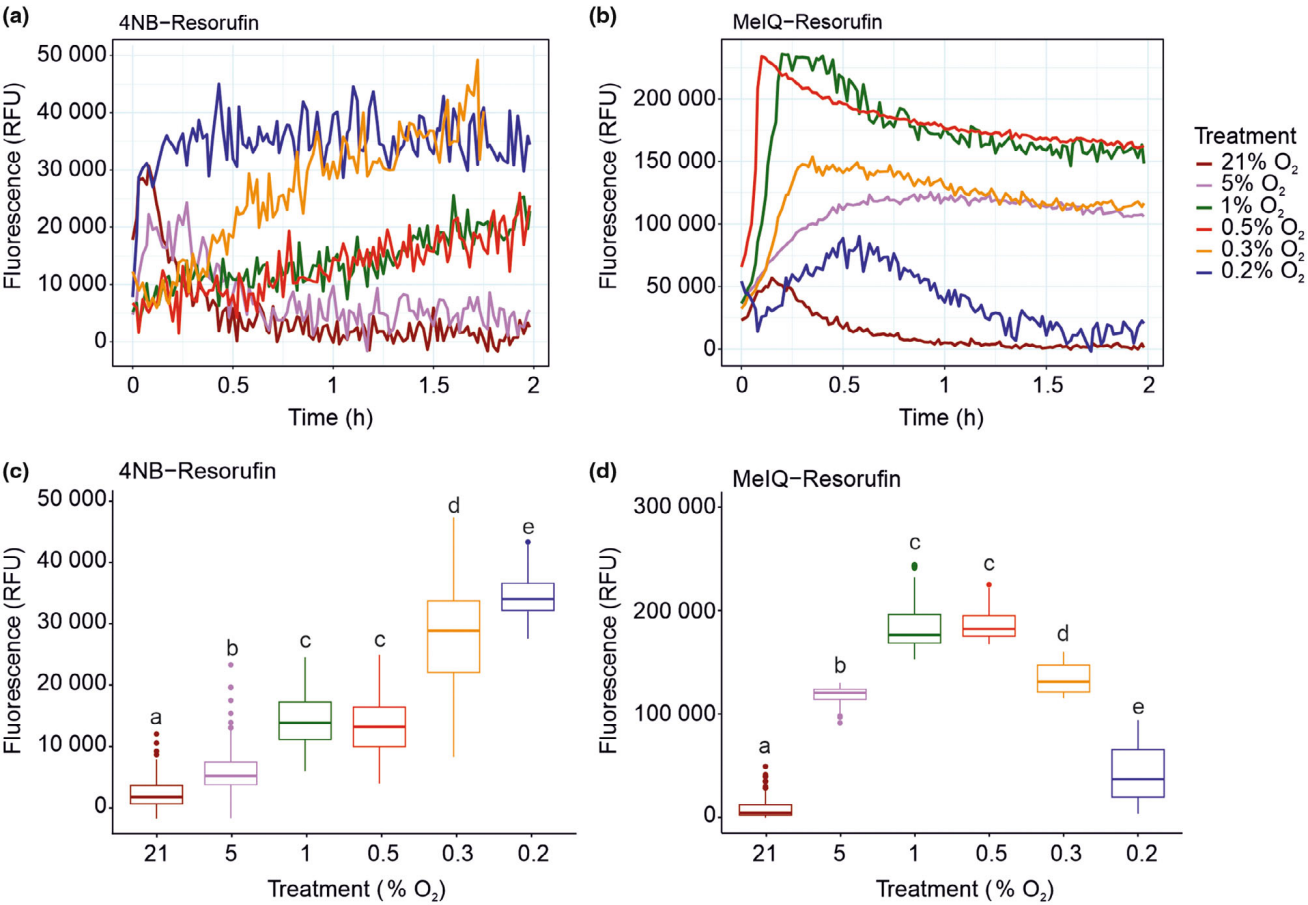
Fluorescence intensity of bioreduced probes varies with O_2_ concentration. Hypoxic incubation of *Arabidopsis thaliana* cells with 4NB‐Resorufin (a) or MeIQ‐Resorufin (b) shows variation in O_2_‐dependent activation of fluorescence with time. Averaged fluorescence intensity (across the duration of 15–120 min) shows a difference in the O_2_ concentration at which the most intense signal is achieved for 4NB‐Resorufin (c) and MeIQ‐Resorufin (d). In all experiments, 1 ml *A. thaliana* whole cells were incubated with 10 μM of the probes at varying O_2_ concentrations. Data in boxplots (c) and (d) were taken from ≥ 3 independent experiments; center lines show median values, boxes indicate the interquartile range (25^th^–75^th^ percentiles), whiskers extend to the maximum and minimum values within 1.5x the interquartile range. Data points further than that distance were considered outliers and marked with points beyond the whiskers; dots indicate individual values. One‐way ANOVA followed by the Tukey HSD *post hoc* test revealed that fluorescence intensity was significantly different from that measured in normoxia in all cases (letters indicate statistical differences between groups, *P*‐value < 0.001).

We next wanted to determine whether the probes could be exploited to detect cellular hypoxia in a higher throughput plate‐based format by measuring fluorescence intensity, and whether such an assay can be used to quantify fluorescence levels as a function of O_2_ concentration. By exposing cells to different concentrations of O_2_, we also wanted to determine whether bioreduction and probe fluorescence were the same for both probes. One millilitre of autotrophic *A. thaliana* 4‐d‐old cell cultures was transferred to 12‐well plates and incubated for 2 h in a plate reader where the atmospheric environment was controlled at concentrations of O_2_ ranging from 0.2% to normoxia (21% O_2_). Plates were kept in dark conditions throughout the experiment to avoid O_2_ production through photosynthesis; therefore, glucose was added to the culture medium to maintain cells in a heterotrophic state. Fluorescence intensity was measured over time for each condition (Fig. 3a,b) and average intensity (from 15 to 120 min) was used to determine overall fluorescence levels (Fig. 3c,d) at each O_2_ concentration.

As detected using microscopy, resorufin fluorescence was observed when cells were equilibrated with both probes in hypoxia compared with cells exposed to normoxia. Cells incubated with 4NB‐Resorufin at O_2_ concentrations of 1% and below showed a sustained increase in fluorescence over the course of 2 h; when the O_2_ concentration was 0.2%, a maximum and sustained level of fluorescence was observed after *c*. 15 min (Fig. 3a). There was an overall increase in fluorescence intensity as O_2_ concentration reduced from 1% to 0.2% (Fig. 3c). When cells were incubated with MeIQ‐Resorufin, a more rapid increase in fluorescence was observed at all O_2_ concentrations measured except 21% (Fig. 3b). The absolute levels of fluorescence attained appeared to be dependent on O_2_ concentration, with the highest levels of fluorescence observed for 1% and 0.5% O_2_ (Fig. 3d). This fluorescence was not sustained, however, and over the course of 2 h, fluorescence levels decreased, most notably in the presence of 0.2% O_2_.

MeIQ‐Resorufin‐derived fluorescence appeared to be detected at higher O_2_ concentrations compared with fluorescence observed in the presence of 4NB‐Resorufin at equivalent O_2_ levels (Fig. 3b,d), consistent with the comparison between these probes in mammalian cells (Wallabregue *et al*., 2023), suggesting that MeIQ‐Resorufin may be capable of detecting milder hypoxia than 4NB‐Resorufin. However, absolute levels of fluorescence were approximately fivefold higher in the presence of MeIQ‐Resorufin than of 4NB‐Resorufin, which may reflect a greater uptake of this probe into the cell, a faster intracellular diffusion or a greater propensity for MeIQ‐Resorufin bioreduction in the *A. thaliana* cell environment. In line with this, the observed decline in fluorescence signal with time for MeIQ‐Resorufin suggests that it is sequestered more rapidly: Resorufin fluorescence is quenched by acidic conditions (pH 6.5 and below; Fig. S4), suggesting that the decline in fluorescence could be a result of vacuolar sequestration of high levels of resorufin derived from the MeIQ‐conjugated probe. Alternatively, resorufin fluorescence quenching could derive from reduced intracellular pH associated with lactate fermentation under hypoxia (Wagner *et al*., 2019). Overall, the results indicate that the probes are capable of detecting intracellular hypoxia in a semi‐quantitative manner in a plate‐based assay, thus extending their utility as hypoxia‐sensing probes.

### 4NB‐Resorufin and MeIQ‐Resorufin can detect hypoxic conditions in *Arabidopsis thaliana* seedling tissues

To confirm the biological relevance of 4NB‐ and MeIQ‐Resorufin as hypoxia probes, we next sought to determine whether cell permeability and bioreduction of the probes could be observed in *A. thaliana* whole seedlings under hypoxic conditions. We anticipated that additional incubation time would be required for diffusion and uptake of the probe in seedlings compared with that in cells; given we also observed a decrease in resorufin fluorescence in whole cells after prolonged incubation with MeIQ‐Resorufin at low O_2_ concentrations, we first determined whether a stable fluorescence signal could be observed in seedlings incubated with 4NB‐ and MeIQ‐Resorufin at severe hypoxia (0.1% O_2_) over the course of 6 h. The fluorescent signal intensity was not strong in cultured cells (Fig. 2); therefore, we increased the probe concentration in these experiments to 100 μM to ensure adequate signal intensity in a system with a more complex tissue structure (including potential barriers to uptake). The arising data (Fig. S5) revealed a time‐dependent increase in resorufin fluorescence intensity over 6 h, suggesting that the probes were sufficiently stable to elicit a detectable signal in the whole‐seedling environment.

**Fig. 4:**
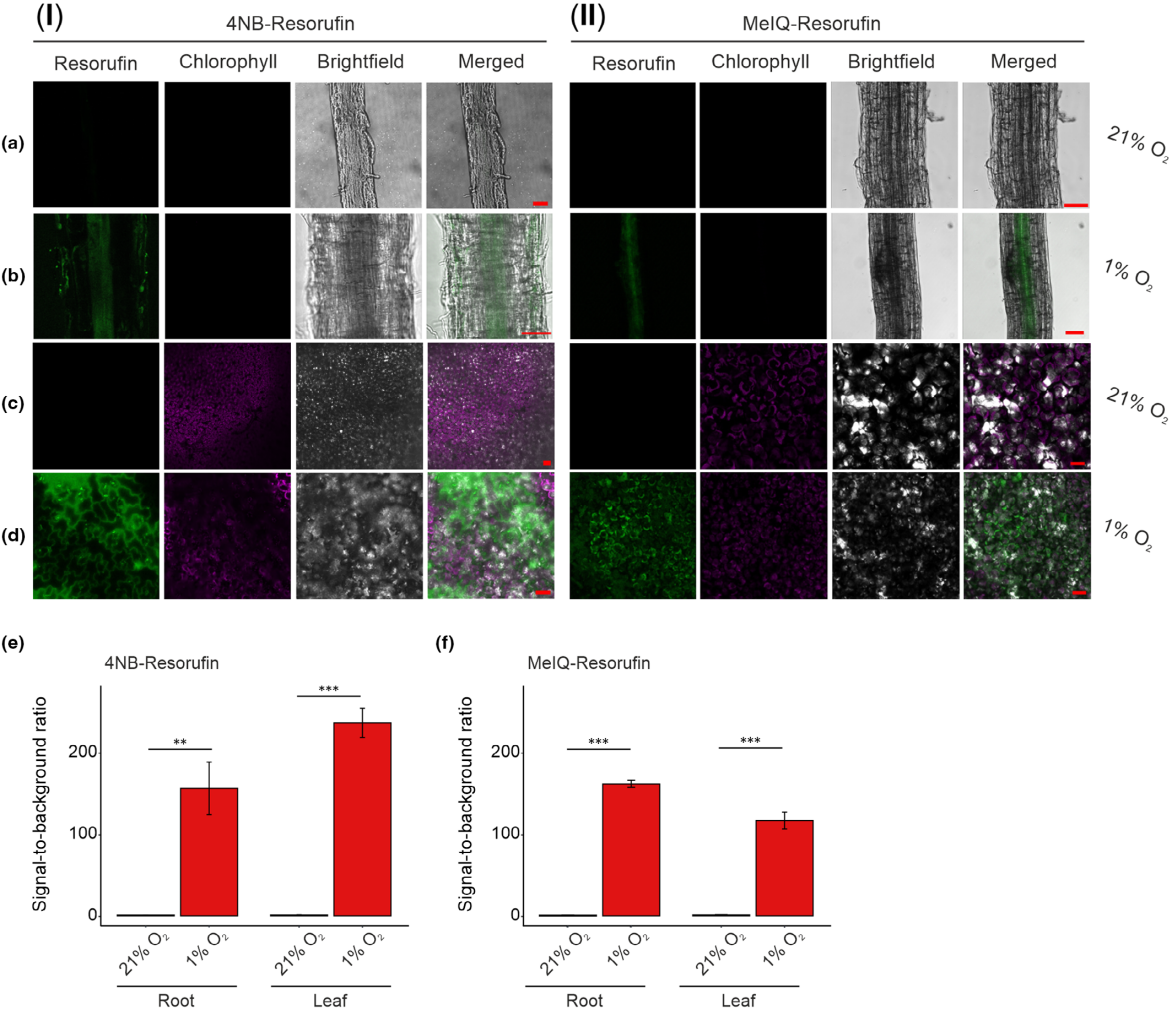
Bioreductive hypoxia‐responsive chemical probes show fluorescence in hypoxic tissue of Arabidopsis. Two‐week‐old Arabidopsis seedlings were incubated with (I) 4NB‐Resorufin or (II) MeIQ‐Resorufin for 6 h under normoxic (21% O_2_) or hypoxic (1% O_2_) conditions. The live root (a, b) and leaf (c, d) of the seedlings were imaged using a confocal (Zeiss LSM880/LSM780) microscope using 561 nm laser to excite and fluorescence of the resorufin measured at 570–600 nm (green) as well as Chl autofluorescence measured at 650–750 nm (purple). The leaf tissue experiments in panel (I) (c) and (d) were performed with 50 μM 4NB‐Resorufin, otherwise 100 μM probes used. Bars, 50 μm. (e, f) Quantification of resorufin fluorescence intensity in seedlings treated with 4NB‐ or MeIQ‐Resorufin upon hypoxia or normoxia represented by the ratio of the signal intensity to the background (*n* = 3). Error bars show standard deviation; statistical significance was determined using two‐sided Student's *t*‐test (**, *P*‐value < 0.01; ***, *P*‐value < 0.001).

We therefore proceeded to examine seedling tissues exposed to normoxia (21% O_2_) or hypoxia (1% O_2_) by confocal microscopy following incubation with both probes (Fig. 4). When seedlings were incubated with 4NB‐Resorufin (Fig. 4(I)), hypoxia‐dependent fluorescence was observed in root tissue (Fig. 4(I)a,b) and leaves (Fig. 4(I)c,d). In the root tissue, fluorescence was observed in the cortex and vascular tissue; in the leaf tissue, fluorescence was observed, albeit not homogenously. Overall, these images indicate that 4NB‐Resorufin was able to permeate the seedling tissue and translocate through the plant, likely via the vasculature. MeIQ‐Resorufin was similarly able to permeate seedling tissue and fluoresce in a hypoxia‐dependent manner. MeIQ‐Resorufin‐derived fluorescence was observed in vascular regions of the root and near‐homogenously in the leaf tissue (Fig. 4(II)a–d). These data suggest translocation of the probe throughout the plant tissue, including to the leaf mesophyll layer. As observed in cells, a significant increase in fluorescence was only observed in the presence of either probe (and hypoxia; Fig. 4e,f); seedlings treated with equivalent volumes of DMSO did not demonstrate fluorescence at 570–590 nm in either hypoxia or normoxia, while Chl autofluorescence was observed in these seedlings in leaf tissue (Fig. S6).

To confirm that seedling intracellular O_2_ concentrations were hypoxic under the experimental hypoxia settings, we employed an *A. thaliana* line containing a luciferase reporter cassette under the control of the HRPE. This HRPE is known to promote ERF‐VII‐mediated gene upregulation under hypoxic conditions (Gasch *et al*., 2016; Akter *et al*., 2024). When seedlings were exposed to normoxia or hypoxia under the experimental conditions used for probe incubation, we detected a significant difference in luciferase activity, commensurate with elevated luciferase expression in hypoxia (Fig. S7), confirming that the seedlings were experiencing a hypoxic environment.

We next checked whether incubation of seedlings with the probes could affect plant growth. Seven‐day‐old Arabidopsis seedlings were treated with 100 μM 4NB‐Resorufin, MeIQ‐Resorufin or resorufin (Fig. S8). No treatment, or treatment with 10% DMSO, was used as negative controls. Physiological measurements were taken after 4 d of the treatment, during which seedlings were kept under 16 h : 8 h photoperiod conditions, in air. Consistent with our results for cell culture, we did not observe any apparent phenotypical differences between seedlings treated with the probes compared with the DMSO negative controls (Fig. S8a). No statistically significant differences were measured in primary root length, growth rate and fresh weight compared with DMSO‐treated seedlings, albeit the comparison with untreated seedlings indicates a mild negative effect due to DMSO (Fig. S8b–d). These results suggest, therefore, that 4NB‐Resorufin, MeIQ‐Resorufin and their reduced form do not affect biomass or root growth in Arabidopsis, in the conditions used in this study. Collectively, these results demonstrate that the probes are capable of acting as fluorescent markers of hypoxia in live plant tissue as well as in cells.

### MeIQ‐Resorufin can detect endogenous hypoxia in *Arabidopsis thaliana* root lateral primordia

**Fig. 5:**
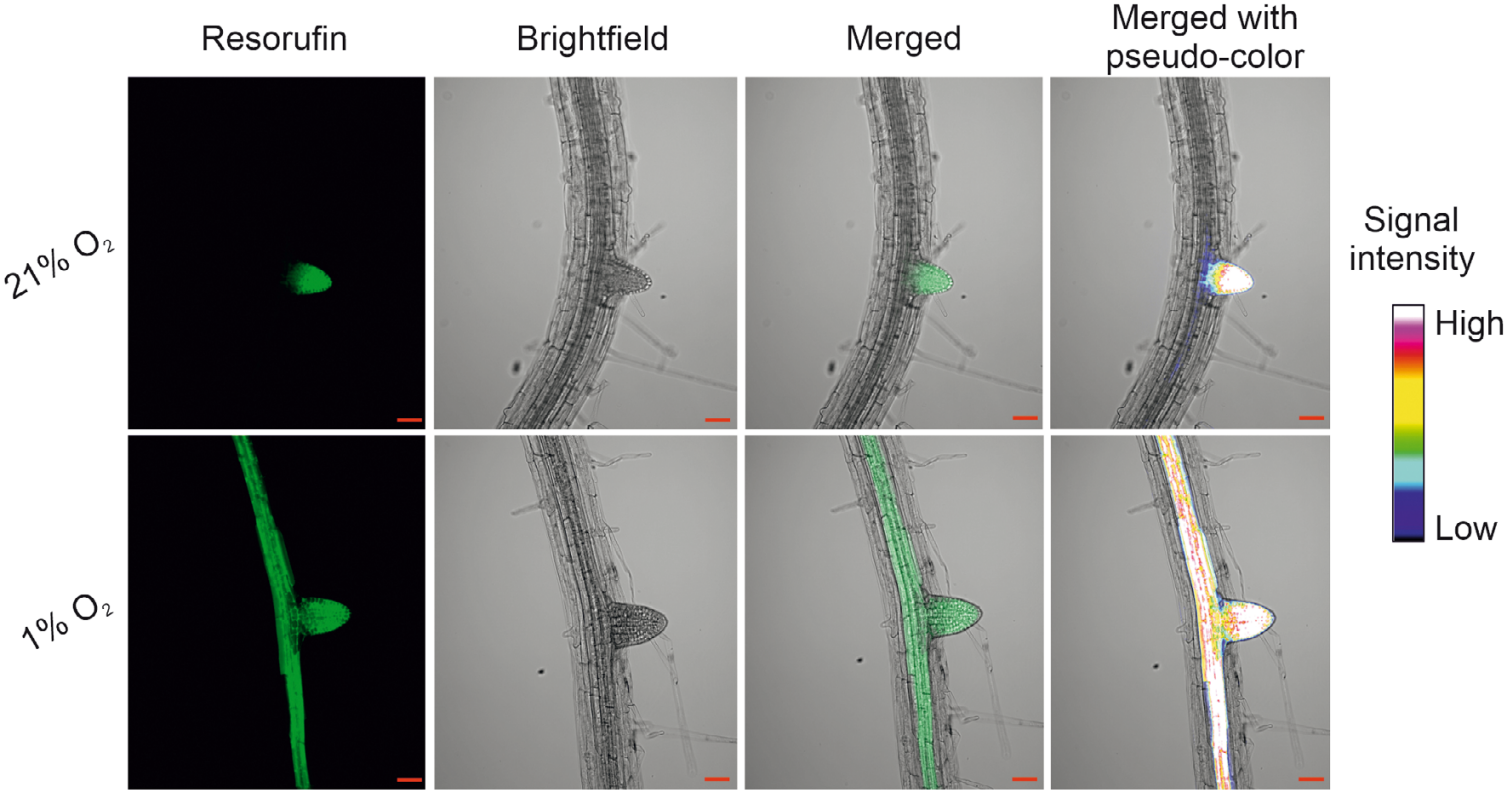
Bioreductive hypoxia‐responsive chemical probes show fluorescence in hypoxic tissue of Arabidopsis. Seven‐day‐old seedlings were incubated with 100 μM MeIQ‐Resorufin for 2 h in normoxic (21% O_2_ v/v) or hypoxic (1% O_2_ v/v) environments. MeIQ‐Resorufin fluorescence was imaged using a Zeiss LSM800 Airyscan laser scanning confocal microscope with 561 nm excitation laser light and 565–616 nm emission detection. Bars, 50 μm; signal intensity bar refers to pseudocolour.

Finally, we sought to determine whether these probes could be used to detect physiologically relevant hypoxia in an otherwise normoxic environment. Arabidopsis root lateral primordia represent a physiological hypoxic niche, arising due to rapid cell division and high metabolism, and demonstrated by localised ERF‐VII stabilisation and hypoxic‐response gene upregulation (Shukla *et al*., 2019). We tested whether MeIQ‐Resorufin could detect hypoxia in root lateral primordia by incubating 7‐d‐old Arabidopsis seedlings with 100 μM probe for 2 h under either normoxic or hypoxic condition. We selected to use MeIQ‐Resorufin for this experiment given the higher levels of fluorescence observed for this probe in the whole cell cultures. Under normoxic conditions, MeIQ‐Resorufin‐derived fluorescence was not typically observed in primary root tissue, as expected (Fig. 4), while fluorescence was consistently observed in root lateral primordia regions at all stages of development (Figs 5, S9). When these tissues were incubated in hypoxia, fluorescence was usually observed in all regions of the root, including the lateral primordia (Figs 5, S9), consistent with previous experiments. These results confirm that the MeIQ‐Resorufin probe can be used to visualise regions of hypoxia in otherwise normoxic Arabidopsis tissues.

## Discussion

The hypoxia‐sensing fluorescent probes 4NB‐Resorufin and MeIQ‐Resorufin are capable of being reduced under hypoxic conditions to release fluorescent resorufin, both *in vitro* and in bacterial and mammalian cells (Collins *et al*., 2018; Wallabregue *et al*., 2023). In this work, we have shown that these probes are also capable of being used to detect hypoxic conditions in plant cells, specifically the model plant *A. thaliana*. Moreover, we were able to demonstrate that the probes can also be applied to *A. thaliana* seedlings and fluoresce exclusively in hypoxia, both when it is exogenously applied (1% O_2_) and endogenous hypoxia, which exists in rapidly metabolising lateral root primordia. Normoxic tissues remained nonfluorescent. This work demonstrates that hypoxia‐sensitive bioreductive enzymes must be present and active in the plant cells and tissues examined, similar to those in other kingdoms (Elmes, 2016; Cenas *et al*., 2021) in order to result in the release of fluorescent resorufin. We cannot rule out that transport dynamics, diffusion barriers, metabolism or other factors affect the homogeneity of probe distribution in seedlings and would therefore recommend interpreting relative intensities of fluorescence in seedling tissues with caution. Indeed, the distribution of each probe may differ due to their differing structures. This may explain why MeIQ‐Resorufin‐derived fluorescence is observed only in root vascular tissue (known to be most susceptible to hypoxic conditions; Armstrong *et al*., 1994; Zabalza *et al*., 2008; Colmer *et al*., 2020), while 4NB‐Resorufin‐derived fluorescence can also be observed in cortical cells. Further studies are necessary to provide detailed information that correlate probe properties with distribution characteristics, particularly in tissues with internal barriers to diffusion.

We were also able to measure probe‐derived fluorescence intensity in a plate‐based format. Here, the use of whole cell cultures reduces issues of homogenous probe distribution and also enables higher throughput analyses that correlate fluorescence intensity with O_2_ availability. However, this was dependent on which probe was used: MeIQ‐Resorufin‐derived fluorescence was significant at 5% O_2_, in contrast to fluorescence derived from 4NB‐Resorufin at the same O_2_ concentration. This indicates that MeIQ‐Resorufin is able to detect ‘mild’ hypoxia (5% O_2_ to 0.5% O_2_) in contrast to 4NB‐Resorufin, which gave significant fluorescence values at 1% O_2_ and below. The mild hypoxia‐dependent response of MeIQ‐Resorufin is consistent with the hypoxia‐sensitivity results observed for this probe in mammalian A549 lung cancer cells and HCT116 colorectal cancer cells spheroids (Wallabregue *et al*., 2023). In these systems, MeIQ‐Resorufin begins producing significant fluorescence at 4% O_2_, where a fivefold increase compared with normoxic controls was observed after 2 h of incubation. Fluorescence increase further intensifies at lower oxygen concentrations with a maximum of 30‐fold increase at < 0.1% O_2_ compared with normoxic controls. In this study, the overall fluorescence levels derived from MeIQ‐Resorufin were approximately fivefold higher than those from 4NB‐Resorufin. Time‐course experiments conducted in *Arabidopsis thaliana* whole plants and cell lysates at 0.1% O_2_ further highlight these differences (Figs S5, S10): MeIQ‐Resorufin exhibited a significant hypoxia‐induced fluorescence increase, with 134‐fold and 157‐fold enhancements compared with the DMSO control in plant cell lysates with and without protease inhibitor cocktails, respectively, after 100 min. Similarly, a 196‐fold increase was observed in whole plants after 360 min. By contrast, 4‐NB‐Resorufin showed only modest fluorescence increases under the same conditions, with 1.6‐fold and 1.7‐fold increases in cell lysates with and without protease inhibitor cocktails, respectively, and a twofold increase in whole plants compared with the DMSO control. Notably, fluorescence increases between the two probes differed significantly, with 117‐fold and 59‐fold higher activation for MeIQ‐Resorufin in plant cell lysates without protease inhibitor cocktail and whole plants, respectively. These findings clearly demonstrate that the differences in activation are predominantly due to the inherently higher bioreductive reactivity of the indolequinone group than that of the 4‐NB benzyl group, although a minor contribution from probe uptake cannot be excluded.

In hypoxic mammalian cells, bioreduction in MeIQ‐Resorufin is rapid (Wallabregue *et al*., 2023), which aligns with the observed higher fluorescence derived from MeIQ‐Resorufin than 4NB‐Resorufin in hypoxic plant cells. Notably in cell cultures, the high levels of fluorescence observed with MeIQ‐Resorufin were not stable over time, with decreases in fluorescence being observed at low O_2_ (< 0.5% O_2_) concentrations. This may be due to acid‐quenching of resorufin fluorescence (Fig. S4), either in mildly acidic conditions associated with hypoxia in the cytosol (Felle, 2005) or due to vacuolar xenobiotic compartmentalisation (Coleman *et al*., 1997), where pH levels will likely lead to a significant decrease in fluorescence. Despite the observation of this effect in whole cells, MeIQ‐derived resorufin fluorescence showed prolonged stability in tissues, which may reflect improved tolerance of hypoxia‐induced metabolic acidosis in tissues compared with that in isolated whole cells (Felle, 2005). Importantly, the ability of MeIQ‐Resorufin to detect mild hypoxia with relatively high signal intensity enabled its use for the detection of endogenous hypoxia in lateral root primordia and may make this the most practical of the two probes for future use *in planta*.

The probes themselves are not significantly bioreduced in the presence of reactive oxygen species (Figs S11, S12), which are known to be elevated in prolonged hypoxia (Jethva *et al*., 2023). However, they are susceptible to prolonged photobleaching and high concentrations of NaCl (Figs S13, S14), so care should be taken in choosing conditions for their application. It is also important to note that bioreduction in these probes is irreversible, so they are not able to indicate real‐time oxygen dynamics; development and application of reversible oxygen probes in plants would be of significant interest (Takahashi *et al*., 2012; Zhang *et al*., 2021). Nevertheless, the bioreductive ability of these chemical probes to detect and stain for hypoxic conditions in plant cells and tissue will be a valuable and timely addition to the suite of techniques available to measure O_2_ availability in the field of plant hypoxia research, with respect to both acute hypoxia (as an environmental stress) and chronic hypoxia (as a developmental cue). They can be applied without the requirement for genetic engineering of biosensors or the challenging aspects of probe measurement (including tissue damage and handling challenges; Weits, 2021). A summary of the properties we observed for each probe in *A. thaliana* cells and seedlings can be found in Table S1; determination of probe permeability and hypoxia‐activation in crop plants will expand their utility. Furthermore, the inference that bioreductive enzymes are present and active in Arabidopsis cells indicates that the same principle of bioreduction could be used to trigger hypoxic activation of other molecules, a principle that has been exploited in mammalian hypoxia biology (Collins *et al*., 2018) and that may enable intervention in a range of situations where hypoxia is relevant.

## Competing interests

None declared.

## Author contributions

MP, MSK, ALDW, VV and AMR conducted experiments and analysed data, assisted by ENS and HB. EF, SJC and EMH conceived the research. EF and DAW designed and supervised the research. ALDW designed the chemical experiments, and SJC supervised the chemistry aspects of the research. SJC supervised the chemistry aspects of the research. MP, MSK, ALDW and EF wrote the manuscript with assistance from all co‐authors. MP, MSK and ALDW contributed equally to this work.

## Disclaimer

The New Phytologist Foundation remains neutral with regard to jurisdictional claims in maps and in any institutional affiliations.

## Supporting information


**Methods S1** General experimental techniques plus different *in vitro* assays, synthetic procedures and HPLC chromatograms for the preparation of 4NB‐Resorufin and MeIQ‐Resorufin.
**Fig. S1** Additional images of *Arabidopsis thaliana* cells incubated with 4NB‐Resorufin and MeIQ‐Resorufin.
**Fig. S2** Fluorescence at 570–590 nm is resorufin‐dependent and conditionals on cellular uptake.
**Fig. S3** 4NB‐Resorufin and MeIQ‐Resorufin are not toxic to *Arabidopsis thaliana* cells and seedlings under the conditions used.
**Fig. S4** Resorufin, MeIQ‐Resorufin and 4NB‐Resorufin fluorescence is quenched at mildly acidic pH.
**Fig. S5** MeIQ‐Resorufin and 4NB‐Resorufin bioreduction in *Arabidopsis thaliana* seedlings at 0.1% O_2_.
**Fig. S6** Hypoxic fluorescence in *Arabidopsis thaliana* tissues is a result of incubation with 4NB‐/MeIQ‐Resorufin.
**Fig. S7** *A. thaliana* seedlings elicit a typical hypoxic response after 1 h incubation in hypoxia (1% O_2_).
**Fig. S8** 4NB‐Resorufin, MeIQ‐Resorufin and their reduced form Resorufin do not affect growth in Arabidopsis seedlings.
**Fig. S9** Additional images of MeIQ‐Resorufin‐derived fluorescence in *Arabidopsis thaliana* lateral root primordia, including at different stages of development.
**Fig. S10** MeIQ‐Resorufin and 4NB‐Resorufin bioreduction in *Arabidopsis thaliana* cell lysates under hypoxia.
**Fig. S11** Stability of 4NB‐Resorufin under treatment with hydrogen peroxide or hydroxyl radical.
**Fig. S12** Stability of MeIQ‐Resorufin under treatment with hydrogen peroxide or hydroxyl radical.
**Fig. S13** Resorufin, MeIQ‐Resorufin and 4NB‐Resorufin fluorescence is quenched by after light irradiation.
**Fig. S14** Resorufin, MeIQ‐Resorufin and 4NB‐Resorufin fluorescence is quenched in buffer with high salt concentrations.
**Table S1** Comparison between chemical probe properties.
Please note: Wiley is not responsible for the content or functionality of any Supporting Information supplied by the authors. Any queries (other than missing material) should be directed to the *New Phytologist* Central Office.

## Data Availability

Data acquired in this study are available in the Supporting Information of this article (Figs S1–S14; Table S1; Methods S1). The time required to synthetically prepare each probe is given in the Methodology section. Requests for resources (including 4NB‐Resorufin and MeIQ‐Resorufin) can be made to the corresponding author.
